# Case Report: Osimertinib-induced acute interstitial lung disease

**DOI:** 10.3389/fphar.2025.1608733

**Published:** 2025-06-05

**Authors:** Zhiwu Lin, Liang Wu, Yang Yu, Jiudong Jiang, Yanchun Yang, Guibao Xiao

**Affiliations:** ^1^ Department of Thoracic Surgery, Ziyang Central Hospital, Ziyang, China; ^2^ Department of Infectious Diseases, Ziyang Central Hospital, Ziyang, China

**Keywords:** adverse drug reaction, osimertinib, interstitial lung disease, lung cancer, EGFR-TKI

## Abstract

Osimertinib is a third-generation irreversible epidermal growth factor receptor tyrosine kinase inhibitor (EGFR-TKI) that selectively targets EGFR-TKI-sensitive mutations, thereby inhibiting tumor cell proliferation, migration, and invasion. Herein, we present a case of osimertinib-induced interstitial lung disease (ILD) in an 80-year-old woman with EGFR-mutated lung adenocarcinoma. The patient was treated with osimertinib as first-line therapy for metastatic non-small cell lung cancer (NSCLC). On day 45 of treatment, she experienced acute onset of severe dyspnea, which rapidly progressed to diffuse bilateral pulmonary consolidation and profound hypoxemia. Despite discontinuation of osimertinib and administration of aggressive supportive care, her clinical condition continued to deteriorate, ultimately resulting in a fatal outcome. This case underscores the importance of monitoring respiratory symptoms in patients receiving EGFR-TKIs, promptly diagnosing ILD, and implementing early intervention to mitigate adverse outcomes.

## 1 Introduction

Lung cancer remains the leading cause of cancer-related mortality worldwide (Siegel et al., 2014). Non-small cell lung cancer (NSCLC) constitutes approximately 80%–85% of all lung cancer cases, with lung adenocarcinoma being the most prevalent histological subtype (Wang et al., 2021). A substantial proportion of lung adenocarcinomas harbor activating mutations in the epidermal growth factor receptor (EGFR). Osimertinib, a third-generation EGFR tyrosine kinase inhibitor (TKI), has become a standard first-line treatment for patients with metastatic NSCLC harboring EGFR exon 19 deletions or exon 21 L858R mutations (Isobe et al., 2021). While gastrointestinal symptoms and skin mucosal lesions are the most frequently reported adverse effects, osimertinib-induced ILD is extremely rare yet represents a severe and potentially fatal adverse reaction (Xie et al., 2020). Herein, we report a case of an EGFR-mutated lung adenocarcinoma patient who developed refractory dyspnea and hypoxemia after 1.5 months of osimertinib therapy, ultimately leading to treatment failure and death.

## 2 Case description

**FIGURE 1:**
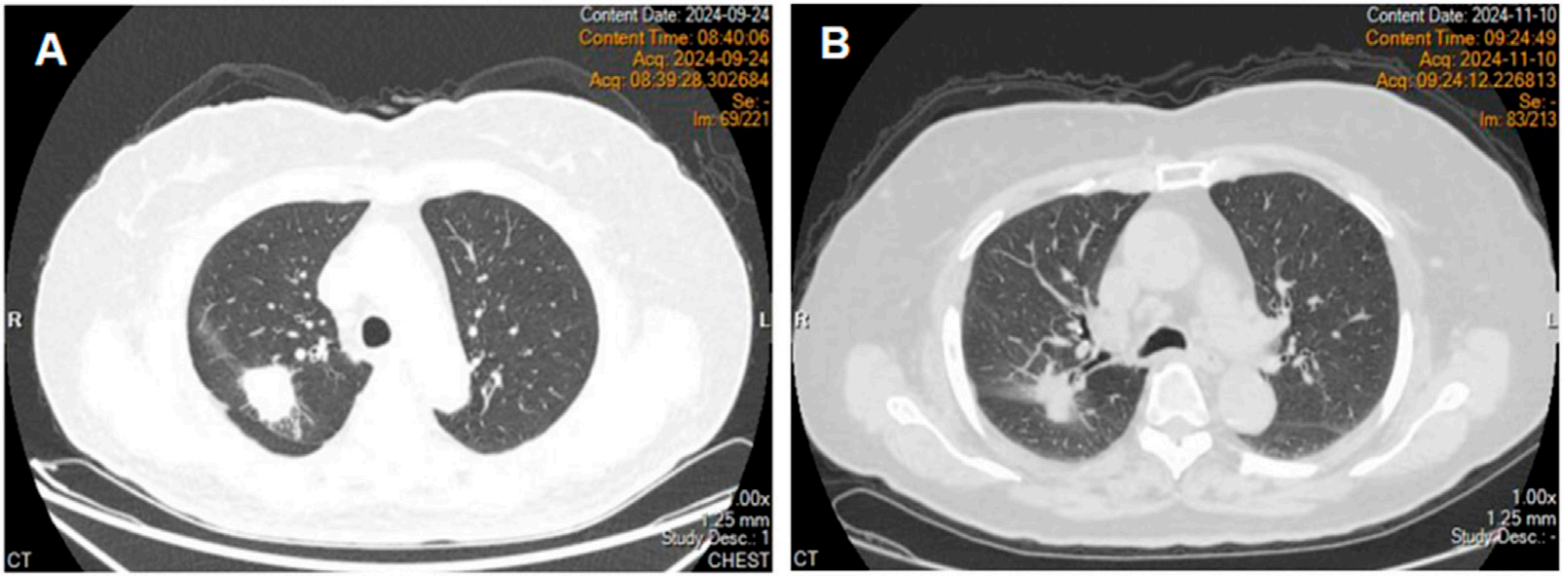
Chest computed tomography revealed a right upper lobe mass **(A)**. Chest computed tomography on day 31 of treatment **(B)**.

An 80-year-old female patient was admitted to the hospital on 24 September 2024, due to a persistent cough. Chest computed tomography (CT) revealed a mass in the right upper lobe with associated obstructive pneumonia (Figure 1A). On October 1, under local anesthesia, a needle biopsy of the lesion was performed, and the pathology confirmed adenocarcinoma. During her evaluation, an enhanced brain magnetic resonance imaging (MRI) scan revealed multiple intracranial metastatic lesions. The patient was subsequently diagnosed with right lung adenocarcinoma with intracranial metastasis (cT2aNxM1c). Gene sequencing identified an EGFR exon 19 deletion mutation. Her medical history included hypertension and cerebral atrophy; however, chronic obstructive pulmonary disease (COPD) or other pulmonary conditions had not been previously documented. Pulmonary function testing upon admission indicated an FEV1/FVC ratio of 86%. Following the diagnosis, she initiated treatment with osimertinib at a dose of 80 mg once daily on October 9.

On day 32 of treatment, the patient developed diarrhea. In response, temporary antidiarrheal medication was administered, leading to improvement in her symptoms. Subsequently, a chest computed tomography was performed, which revealed no abnormalities in the lungs (Figure 1B). The clinical response was assessed as partial remission (PR), and osimertinib treatment was continued.

On the 37th day of treatment, the patient experienced mild shortness of breath after exercise, accompanied by cough and sputum production, primarily characterized by white mucus sputum,and the symptoms were mild.

On the 45th day of treatment, the patient developed resting dyspnea. A physical examination showed an oxygen saturation level of 78% on room air, which increased to 90% with mask oxygen supplementation, accompanied by bilateral lung crackles. Laboratory findings revealed abnormal blood gas values (pH 7.46, PO2 48 mmHg, PCO2 34.7 mmHg) and negative results for respiratory pathogens. However, inflammatory markers, including C-reactive protein, interleukin levels, and neutrophil percentage (NEUT%), were markedly elevated beyond the normal range. Notably, B-type natriuretic peptide (BNP) and D-dimer levels remained within the normal range. Comprehensive assessments of cardiac, hepatic, and renal functions revealed no significant abnormalities. A diagnosis of pulmonary infection complicated by type I respiratory failure was established. Despite receiving oxygen therapy, anti-infective treatment, and hormone-mediated anti-inflammatory therapy, the patient’s clinical condition showed no significant improvement.

**FIGURE 2:**
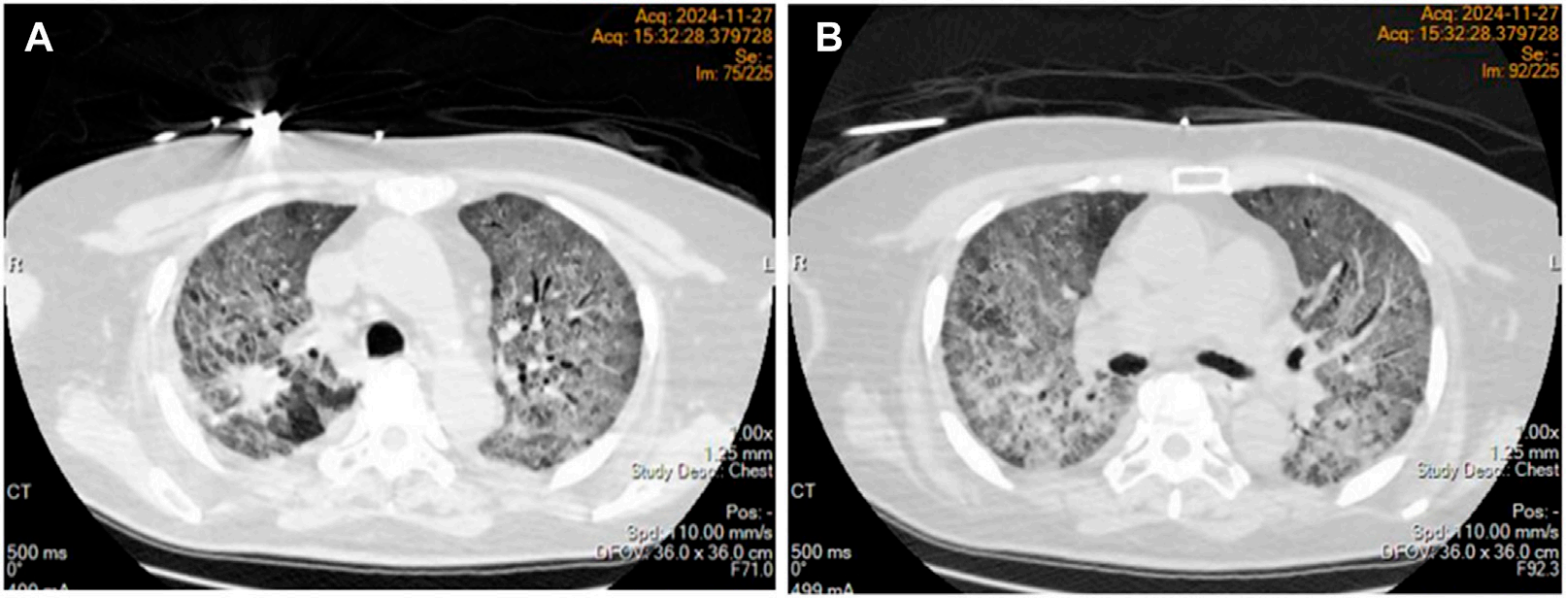
Diffuse interstitial lung disease was present in both lungs **(A,B)**.

On day 48 of treatment, the patient experienced a progressive worsening of dyspnea, necessitating mechanical ventilation. Despite an oxygen concentration exceeding 60%, the arterial partial pressure of oxygen (PaO2) remained at only 40–50 mmHg. Emergency chest computed tomography (CT) revealed diffuse exudative changes in the bilateral lung parenchyma, with involvement exceeding 90% of the lung fields (Figures 2 A, B). Osimertinib was discontinued, and sulprostone was initiated for anti-infective treatment. Additionally, methylprednisolone pulse therapy (500 mg/day) was administered for 3 days, followed by maintenance therapy at 2 mg/kg/day. During this period, the patient developed bilateral spontaneous pneumothorax, which was managed with bilateral thoracic closed drainage.

**FIGURE 3:**
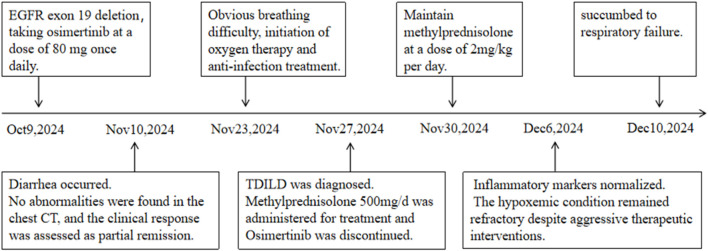
Schematic of the patient’s treatment history.

On the 53rd and 57th days of treatment, C-reactive protein, interleukin, and neutrophil percentage all returned to normal levels. However, clinical symptoms remained unresolved, lung lesions showed no significant improvement, and persistent hypoxemia failed to be corrected. The patient eventually succumbed to respiratory failure on 10 December 2024. Figure 3 presented the patient’s treatment timeline.

## 3 Discussion

Osimertinib is an oral, irreversible, third-generation EGFR tyrosine kinase inhibitor (EGFR-TKI) that exhibits significantly superior efficacy compared with first- and second-generation EGFR-TKIs (Soria et al., 2018). It is currently the standard first-line treatment for advanced non-small cell lung cancer (NSCLC) harboring EGFR mutations. Common adverse effects of osimertinib include diarrhea, rash, and oral mucosal lesions, which are generally well tolerated. However, interstitial lung disease (ILD) represents a rare but potentially fatal pulmonary complication in some patients receiving osimertinib (Fan et al., 2019). ILD encompasses a group of diffuse lung diseases affecting the lung interstitium, alveolar spaces, or bronchioles, often characterized by severe lung function impairment and hypoxemia. Targeted drug-induced ILD (TDILD) is a specific form of ILD caused by targeted therapies (Ma and Dai, 2022). According to the CTCAE classification, TDILD is categorized into four grades (National Institutes of Health, 2025), and its histopathological features typically demonstrate diffuse alveolar damage, which is the primary cause of respiratory failure. The onset pattern of TDILD varies, manifesting as either acute or subacute onset with prominent clinical symptoms that may become life-threatening within a short period, or chronic insidious onset progressing gradually to respiratory failure. The reported incidence of osimertinib-induced interstitial pneumonia is approximately 1%–3%, with a mortality rate of 0.13% (De et al., 2019; Priantti et al., 2024). Compared with phase III clinical trial results for other EGFR-TKIs such as erlotinib and afatinib, osimertinib-associated ILD has a higher mortality rate of approximately 0.4% (Mok et al., 2017). Clinical case reports indicate that TDILD often manifests early during treatment, with a median onset time of 34 days (Zhou et al., 2024). In this case, the patient developed CTCAE Grade 4 interstitial pneumonia 44 days after initiating osimertinib, consistent with the median onset time of 2.7 months noted in the osimertinib mesylate label. The exact pathogenesis of TDILD remains unclear but may involve the role of EGFR in the repair and regeneration of alveolar epithelial cells. Some studies suggest that EGFR-TKIs might interfere with the proliferation and differentiation of type II alveolar epithelial cells (AT2) and inhibit epithelial cell repair following injury by targeting the EGFR signaling pathway in tumor tissues, leading to alveolar structural disruption and fibrosis (Ohmori et al., 2021). In addition, EGFR-TKI can induce chronic inflammation in alveolar and bronchial epithelia, promote the migration and proliferation of fibroblasts, and stimulate the production of extracellular matrix, ultimately resulting in pulmonary fibrosis. Furthermore, these agents may also trigger immune-mediated allergic reactions, contributing to the development of ILD (Matsuno, 2012).

Pathological testing remains a reliable method for confirming TDILD; however, given the challenges in obtaining pathological tissue samples, the diagnosis of TDILD frequently depends on clinical medication history, imaging findings, and the exclusion of other potential causes (Saito and Gemma, 2012; He and Zhou, 2019). In this case, the patient’s condition was critical, and the family declined needle biopsy, rendering pathological evidence unattainable. Nonetheless, based on established clinical diagnostic criteria, the patient demonstrated imaging features consistent with acute interstitial pneumonia, with involvement exceeding 75% of lung parenchyma. Tests for relevant pathogens returned negative results, and cardiogenic causes as well as pulmonary embolism were excluded. Considering the patient’s history of osimertinib treatment, a diagnosis of TDILD (CTCAE grade 4) was confirmed. Currently, the management of TDILD lacks standardization but generally involves discontinuing targeted therapy and suppressing inflammatory responses to control pulmonary interstitial fibrosis. For patients with CTCAE grade 2–4 pulmonary toxicity, it is recommended to discontinue EGFR-TKIs and initiate oxygen therapy and hormone therapy. Although there is no definitive consensus regarding the optimal dose of hormone therapy, the most commonly used regimen is prednisone at 0.5–2 mg/kg/day. For patients with CTCAE grade 4 pulmonary toxicity, methylprednisolone pulse therapy (500–1,000 mg/day) can be administered for up to 3 days to improve the condition (Chinese Anti-Cancer Association Chinese Society of Lung Cancer, 2019). TDILD progresses rapidly, and the mortality rate associated with CTCAE grade 4 is high. Early intervention is crucial for achieving better therapeutic outcomes.

## 4 Conclusion

Herein, we report a series of interstitial lung diseases potentially induced by osimertinib. The underlying mechanisms remain unclear but may involve multiple factors, such as inhibition of the EGFR pathway in normal lung tissue, impaired regeneration and repair processes, and abnormal immune activation. The incidence of ILD caused by osimertinib is significantly lower than that of the skin and digestive tract adverse reactions, but the mortality rate is still high due to the rapid development of the disease and timely discontinuation of the drug. Clinicians should be aware that any new respiratory symptoms in patients using EGFR-TKI should be carefully evaluated and alert for the development of ILD. Early identification and hormone treatment are essential to improve the safety of medication.

## Data Availability

The original contributions presented in the study are included in the article/supplementary material, further inquiries can be directed to the corresponding authors.
